# The Influence of Graphite Filler on the Self-Lubricating Properties of Epoxy Composites

**DOI:** 10.3390/ma17061308

**Published:** 2024-03-12

**Authors:** Jakub Smoleń, Piotr Olesik, Krzysztof Stępień, Marta Mikuśkiewicz, Hanna Myalska-Głowacka, Mateusz Kozioł, Anna Gawron, Marcin Godzierz

**Affiliations:** 1 Faculty of Materials Engineering, Silesian University of Technology, Krasińskiego 8, 40-019 Katowice, Poland; piotr.olesik@polsl.pl (P.O.); krzysztof.stepien@polsl.pl (K.S.); marta.mikuskiewicz@polsl.pl (M.M.); hanna.myalska@polsl.pl (H.M.-G.); mateusz.koziol@polsl.pl (M.K.); 2 Centre of Polymer and Carbon Materials, Polish Academy of Sciences, M. Curie-Skłodowskiej 34 Street, 41-819 Zabrze, Poland; agawron@cmpw-pan.pl (A.G.); mgodzierz@cmpw-pan.edu.pl (M.G.)

**Keywords:** polymer composites, tribology, self-lubricating materials, graphite, sliding materials

## Abstract

In this work, epoxy composites filled with flake graphite of various size (less than 10 μm and less than 45 μm) were produced. The aim of the research was to develop a self-lubricating material with favorable tribological properties, i.e., reduced friction coefficient compared to unfilled epoxy resin and limited abrasive wear. The research material was produced using technical epoxy resins based on bisphenol A. The detailed process of composite production was described, and typical technological problems were considered. The addition of graphite led to an increase in dynamic viscosity, which positively limits the phenomenon of sedimentation, but an increase in the filler content also led to an increase in the porosity of the material. A series of tests have shown that the addition of graphite above 5% by weight allows for a reduction in the friction coefficient from 0.6 to 0.4 and significantly reduces the material’s tendency to abrasive wear.

## 1. Introduction

Polymer composites are a broad group of modern engineering materials. They are used in many industry sectors, including the automotive industry, aviation industry, wind energy industry, shipbuilding industry, production of pipes and transport tanks, etc. The growing demand for materials combining high mechanical strength, low weight and other additional properties (adapted to the area of use) allows for the dynamic development of composites [1].

**Figure 1:**
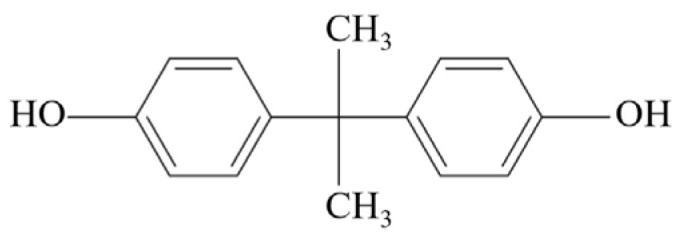
Chemical structure of bisphenol A [3].

One of the most frequently used epoxy resins [2,3] in the production of polymer composites is a resin based on bisphenol A (BPA), the chemical structure of which is shown in Figure 1. Numerous scientific works in the field of composites use pure bisphenol A substrates or ready-made compositions of technical resins, which are modified with, e.g., diluents, flame retardants, UV stabilizers, etc. for easy processing and stability. BPA-based composites, despite concerns about potential toxicity, are used in dentistry to fill cavities and rebuild tooth structure [4,5].

Polymer matrix composites with the addition of graphite are often described in the literature due to the easy availability and low price of this filler. Zhang et al. used graphite and PTFE to produce composites intended for anodes in microbial fuel cells [6]. In turn, Liu et al. [7] tested graphite-reinforced polymers that are resistant to high temperatures and are dedicated to sealing geothermal wells. By adding properly protected graphite to inexpensive rubber, you can increase thermal resistance by up to 80 °C. There are also known applications of composites with the addition of graphite in de-icing materials [8]. However, it seems that the largest number of publications are focused on composites with the addition of graphite, which have favorable tribological properties and contribute to a lower friction coefficient and self-lubricating properties [9].

Self-lubricating materials are an important group of materials with significant technical applications. Self-lubricating materials allow continuous operation without external lubrication, which is why they are often called service-free [10] or sliding materials [11]. The increasing interest in polymer materials and their composites has led to the continuous development of these groups of materials, which allows them to replace traditional materials from the group of metals or ceramics. The phenomenon of widespread use of polymer materials and polymer composites is observed in the sector of self-lubricating materials, including thermoplastics such as polyamide (PA) [12], polyacetal (POM) [13], polytetrafluoroethylene (PTFE) [14], polyether ether ketone (PEEK) [15], polyethylene (PE) [16], polyimide (PI) [17] and others. These polymer materials are often used as unfilled/unreinforced components or composites. Self-lubricating polymer composites are filled with particles of graphite, molybdenum disulfide, PTFE powder, metallic powders, etc., which are mainly intended to reduce the coefficient of friction and increase resistance to abrasive wear [18,19].

Self-lubricating materials are widely used for the production of self-lubricating bearings in the automotive [20], aviation [21], power [22], electronics and household appliances [23] industries. They should meet a number of requirements, the most important of which are resistance to abrasive wear, low friction coefficient, low thermal expansion coefficient and good thermal conductivity [24,25,26,27]. Some limitations in the applicability of self-lubricating materials using polymeric materials may be the non-linearity of the thermal expansion coefficient, creep phenomenon typical of polymers, softening at elevated temperatures, processing shrinkage and hygroscopicity leading to swelling [28,29,30]. Sliding bearings made of solid plastics are limited by production technologies such as injection molding and extrusion, where they must meet specific shape and dimension criteria [31,32].

In self-lubricating polymer composites, the matrix material is responsible for the shape of the product, carrying external loads, protecting the reinforcement material against the unfavorable effects of environmental conditions, and determining thermal and chemical resistance. The reinforcement material is responsible for improving the strength and thermal properties (including reducing the coefficient of thermal expansion), increasing abrasion resistance and stopping the spread of cracks [33,34,35]. The beneficial phenomenon of synergism caused by the coexistence of phases with mutually reinforcing features leads to the improvement of some properties. Depending on the reinforcing phase used, polymer composites can be obtained by many techniques and their modifications. Among the basic methods of production of composites with the use of thermosetting polymers, there are hand lay-up processes [36], spray lay-up processes [37], vacuum bag processes [38], infusion molding technologies [39], including RTM [40], autoclave molding [41], RIM molding [42], hot-pressing [43], as well as pultrusion [44] and casting techniques [45].

The production of thermoset polymer composites where short fibers or granular fillers are the dispersed phase is difficult due to a number of phenomena leading to the occurrence of various problems. The most common problems in liquid resin systems include the following [46,47]:

- Sedimentation of filler particles;
- Porosity of the material;
- Filler particles flotation;
- Inhomogeneity caused by [47] agglomeration of filler particles;
- High crosslinking temperature leading to resin boiling in the volume;
- Limited production time due to crosslinking reactions.

This publication is devoted to the description of the influence of the addition of flake graphite of various particle sizes on the self-lubricating properties of composites and the description of typical problems in the production of polymer composites based on liquid epoxy resins. The research presents the influence of flake graphite with a size below 10 μm and a size below 45 μm on the tribological properties of the produced composite, taking into account graphite weight additions in the range of 5–20%. The tribological properties were tested for the steel material of the counter-sample in reciprocating motion over a distance of 450 m. A number of tests were carried out and basic technological problems were discussed.

## 2. Materials and Methods

**Figure 2:**
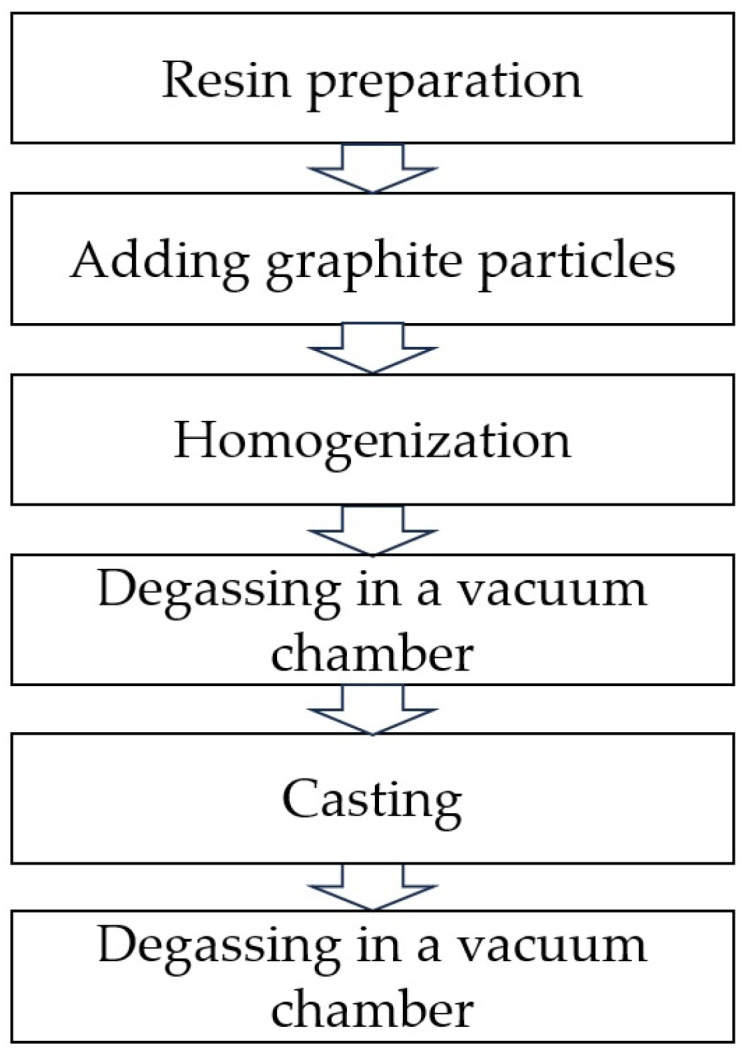
Scheme of the production of composites based on liquid epoxy resin with the addition of flake graphite particles.

To prepare the material samples, an epoxy resin based on bisphenol A LH289 (Havel Composites, Svésedlice, Czech Republic) cross-linked with H135 (Havel Composites, Czech Republic) was used in a weight ratio of 100:35. The scheme for producing samples by casting is shown in Figure 2. The resin and hardener were mixed in appropriate weight proportions, then the planned amount of filler-flake graphite was added to them. The whole thing was homogenized and poured into silicone molds. The mixture after homogenization was placed in a vacuum chamber for degassing at 20 °C. The degassing procedure was repeated also after casting to release any air from the sample [45]. Two types of flake graphite were used: MG 394 with a particle size of less than 45 μm (Sinograf SA, Toruń, Poland) and MG 1596 with a particle size of less than 10 μm (Sinograf SA, Poland). A detailed description of the prepared materials is presented in Table 1.

Dynamic viscosity was measured using a Brookfield DV1 LV viscometer (Brookfield Engineering Laboratories, Middleboro, MA, USA) to determine changes in the resin after adding graphite particles. The composite samples were observed using light microscopy using an Olympus GX71 microscope (Olympus, Shinjuku, Japan). The cross-sectional images were quantitatively analyzed to determine the volumetric share of graphite in the resin and to determine the degree of particle sedimentation using the ImageJ software (version 1.54a). The density and open porosity of the composites were determined using the hydrostatic Archimedes method. The hardness of the samples was determined by measurements on a HK460 hardness tester (Heckert, Leipzig, Germany). Three-point bending strength was performed according to ISO 178 [48] using a Shimadzu AGX-V testing machine (Shimadzu, Kyoto, Japan) with a support spacing of 60 mm at a speed of 5 mm/min.

The dynamic friction coefficient was determined using the “pin-on-block” method using the TM-01M tribotester [46]. Tribological tests were carried out under technically dry friction conditions. The counter-sample material was a steel pin (hardened carbon steel with a hardness of 60 ± 2 HRC, DIN 100Cr6) with a diameter of 6 mm. The pressure of the pin on the sample surface was 1 MPa, reciprocating movement was over a path of 12 mm, total distance was 450 m, and friction speed was 0.1 m/s. The data were presented as a function of friction coefficient vs. distance. The maximum depth of friction paths was measured using the Surftest SJ-400 roughness meter (Mitutoyo, Kanagawa, Japan) and then schemes of cross-sections of tested samples were prepared according to the obtained results.

## 3. Results and Discussion

### 3.1. Cross-Section of Materials and Quantitative Assessment 

**Figure 3:**
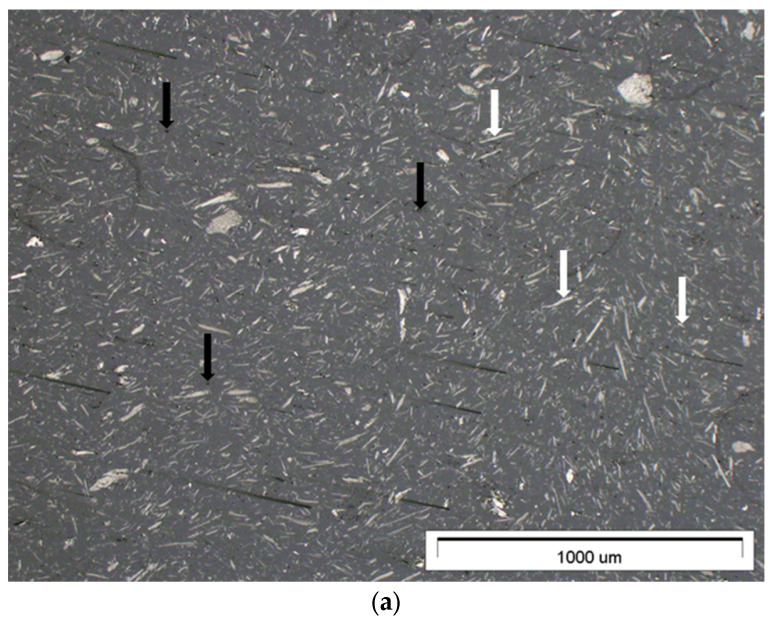

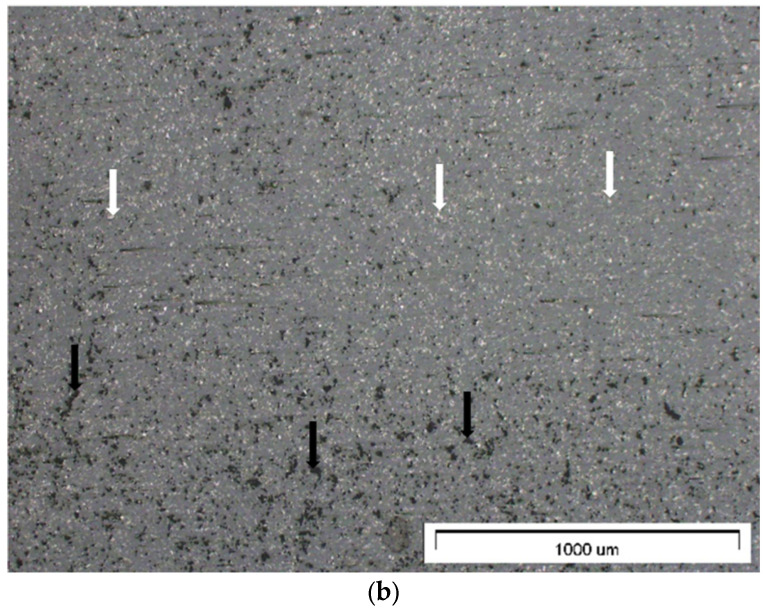
Cross-section of representative samples: (**a**) G45_15; (**b**) G10_15 (optical microscope, 5× magnification). In the figures, white arrows mark graphite particles, and black arrows mark porosities.

Figure 3 shows a cross-section of representative composites containing 15% graphite filler with a particle size of less than 45 μm (Figure 3a) and a particle size of less than 10 μm (Figure 3b).

Both figures show a uniform distribution of filler particles and no agglomerates. A composite containing finer graphite has greater closed porosity, which may have an adverse effect on the mechanical properties of the material. In the figures, the white arrows mark graphite particles, and black arrows mark porosity values. The quantitative image analysis, the results of which are included in Table 2, indicates a similar, proportional surface share of graphite particles for all materials produced, which is beneficial and proves the high homogeneity of the material and the limited phenomenon of particle sedimentation under the influence of gravity in the liquid resin. A detailed description of the procedure for selecting process parameters that ensure the reduction of sedimentation is described in a previous article [47].

**Figure 4:**
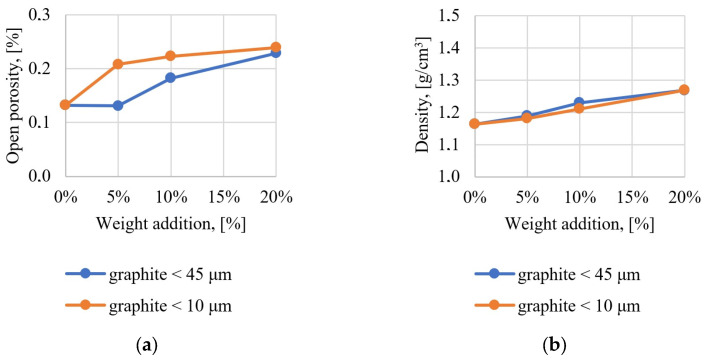
Results obtained using the Archimedes method: (**a**) open porosity; (**b**) material density.

The density and open porosity of the composites were determined using the Archimedes method, the results of which are presented in Figure 4. The addition of flake graphite leads to an increase in the porosity of the composite, which results from technological problems with accurate degassing of the composite. The addition of graphite with a particle size below 10 μm leads to a rapid increase in porosity (about 0.2%), while the addition of a larger amount of graphite in the range of 5–20% does not lead to significant changes. A slightly different character is represented by larger graphite particles with a size below 45 μm, where the 5% addition does not lead to a change in open porosity compared to the unfilled epoxy resin, while increasing the graphite fraction to 5–20% leads to a gradual increase in porosity. Composite samples containing 20% graphite by weight are characterized by similar porosity regardless of the size of graphite particles. The density of the composites increases with the increase in the addition of graphite from a value of 1.16 g/cm^3^ for unfilled epoxy resin to a density of approximately 1.3 g/cm^3^ for composites with 20% graphite addition (the size of graphite particles has no significant impact on the density).

### 3.2. Viscosity

**Figure 5:**
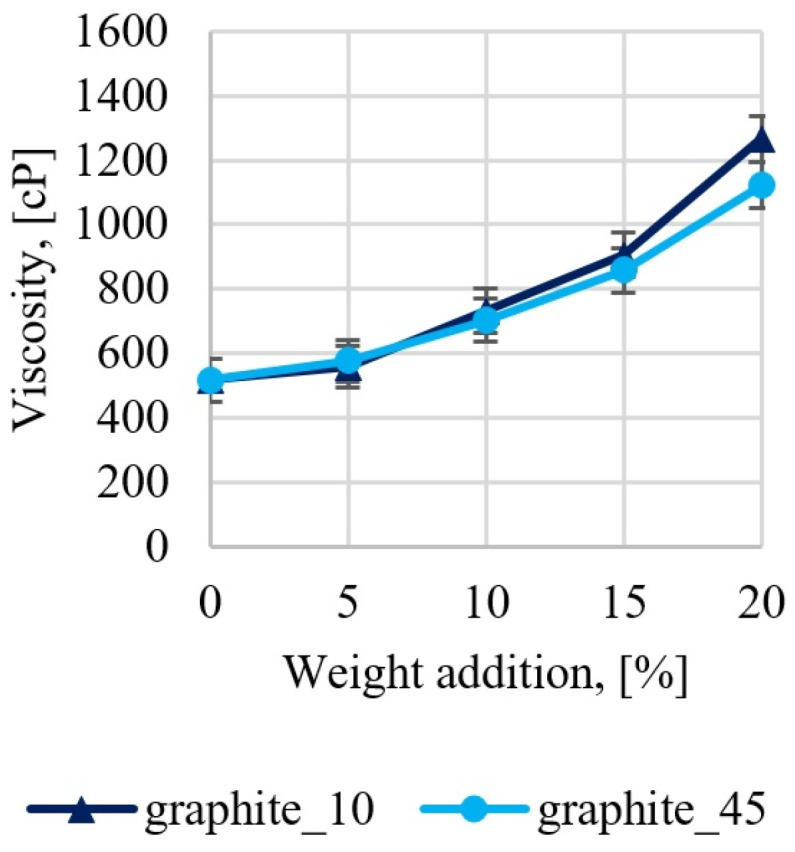
Results of dynamic viscosity measurements determined within 5 min of adding the graphite at 20 °C.

An important technological parameter that determines both the sedimentation rate of graphite particles and the ease of degassing of the system is dynamic viscosity. After adding the hardener, the epoxy resin undergoes complex cross-linking processes during which temperature and viscosity changes occur; therefore, determining the exact viscosity value through continuous measurement may be problematic. The tests assumed that the optimal measurement time is 5 min after adding the hardener and graphite. A more detailed physical and mathematical description of the phenomena and sedimentation parameters were described in a previous publication [47]. The test results are presented in Figure 5. The epoxy resin at a temperature of 20 °C has a viscosity of approximately 500 cP, the addition of graphite leads to an increase in the viscosity of the system, and graphite particles with a smaller diameter lead to a slightly greater increase in the viscosity of the system, which results from the fact that in this same mass of filler, the number of particles is greater when the particles are smaller in size. In addition, smaller particles can hinder polymer chains movement more than larger particles. The differences between graphite sizes below 10 µm (G10 samples) and below 45 µm (G45 samples) are small. The addition of 5% graphite by weight increases the viscosity to 600 cP (20% increase), 10% to approximately 800 cP (60% increase), 15% to approximately 1000 cP (100% increase), and 20% to approximately 1200 cP (140% increase). It was estimated that a dynamic viscosity of 600 cP (5% graphite addition by weight) is sufficient to ensure that the sedimentation processes are minimal, considering a gelation time of the system of approximately 30 min. After reaching the gelation point, the resin has a high viscosity, which stops the sedimentation and flotation process of particles. The addition of graphite above 15% leads to problems with effective degassing of cross-linking systems due to hindering the movement of air bubbles.

### 3.3. Hardness

**Figure 6:**
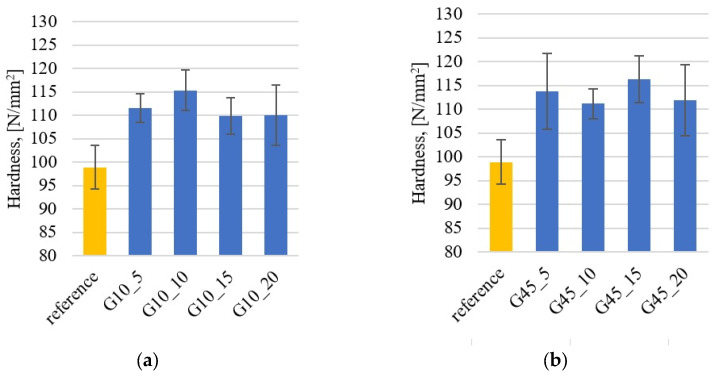
Brinell hardness results for composites with addition of graphite: (**a**) under 10 μm; (**b**) under 45 μm.

Hardness measurements are presented in Figure 6. The unfilled epoxy resin has a hardness of approximately 100 HB, and the addition of graphite increases the hardness by approximately 10–15% on average regardless of the size of graphite and its weight addition (in the range of 5–20%). A similar nature of hardness changes after adding graphite was observed by Albozahid et al. [49] and Shalwan et al. [50], citing the beneficial synergistic effect of the hardness pattern as a result of adding harder graphite particles than epoxy resin [51]. 

### 3.4. Mechanical Properties 

**Figure 7:**
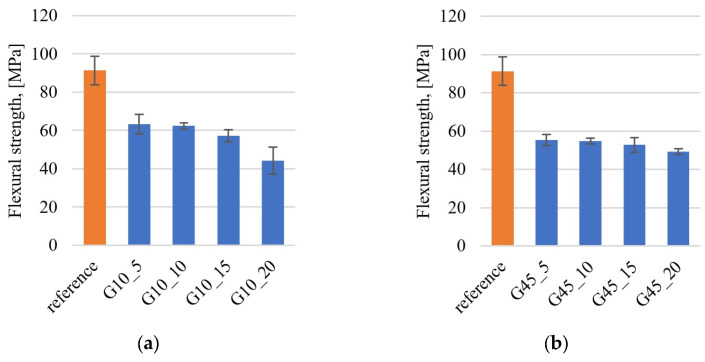
Flexural strength for samples containing graphite: (**a**) under 10 μm; (**b**) under 45 μm.

The test results for three-point bending strength are presented in Figure 7 and Table 3. The addition of graphite leads to a decrease in flexural strength. The strength of the unfilled epoxy resin is approximately 90 MPa, and the addition of 5% graphite leads to a decrease in strength by approximately 30 MPa both in samples with graphite particles below 10 μm and in samples containing graphite below 45 μm. The differences between the G10 and G45 series are insignificant. The greatest decrease in strength was observed in the sample containing 20% of fine graphite (sample G10_20), which is due to the large number of pores in the material, which has a negative impact on the mechanical strength. Young’s modulus does not differ significantly between the reference samples (unfilled epoxy resin) and composite samples containing graphite. The tests simultaneously revealed a positive effect of the addition of graphite on hardness and a negative effect on bending strength, which results from the research methodology, where the small size of pores does not negatively affect the results of hardness measurements using the Brinell method but is of great importance in bending as porosities generate discontinuities in the material, which lead to the destruction of the composite under the influence of lower forces than in the case of the unfilled epoxy resin.

### 3.5. Tribological Properties

**Figure 8:**
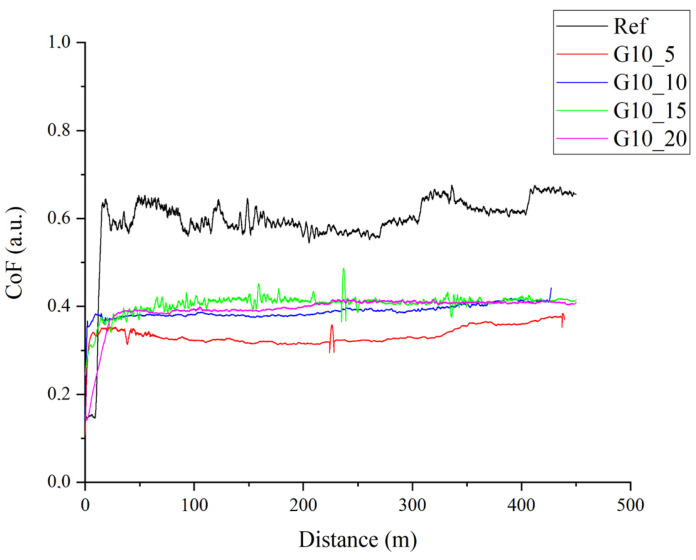
Dynamic coefficient of friction vs sliding distance curves for the composites containing graphite filler at particle diameter below 10 μm.

**Figure 9:**
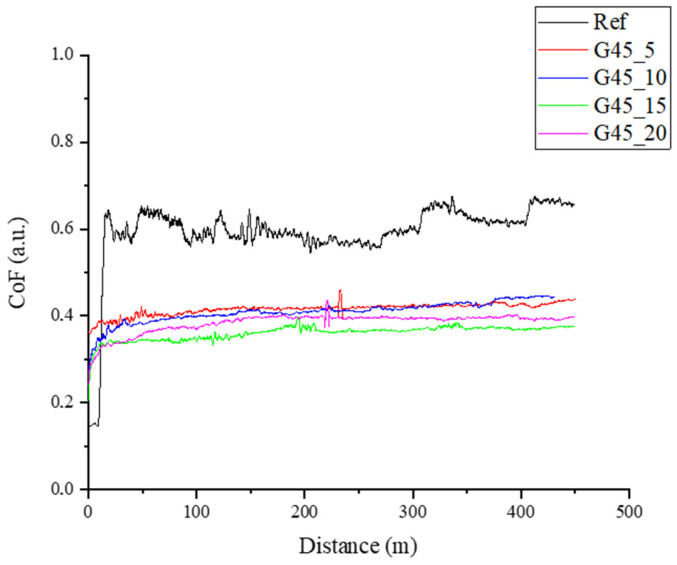
Dynamic coefficient of friction vs. sliding distance curves for the composites containing graphite filler at particle diameter below 45 μm.

The essential part of the research work was the assessment of the tribological properties of the produced composites and the assessment of abrasive wear. The results of tribological tests are presented in Figure 8 and Figure 9 and are included in Table 4. The addition of graphite, regardless of particle size, leads to a similar reduction in the friction coefficient, which for the unfilled epoxy resin is approximately 0.6, and for the tested composites, it is approximately 0.4 (reduction in the friction coefficient by over 30%). The lapping distance for the tested materials is short and is about 20–30 m. The presence of graphite in the composite structure allows to reduce the friction coefficient and reduce abrasive wear due to the formation of a carbon tribofilm on the surface [52,53].

The unfilled epoxy resin sample has the largest volume loss, and the addition of graphite significantly reduces material loss, which extends the service life. This is due to [54,55,56] formation of carbon tribofilm, which effectively protects the wear surface from further abrasive damage. An additional advantage of introducing graphite particles into the epoxy resin is the increase in the thermal conductivity coefficient, which is desirable for friction systems because graphite has the ability to effectively dissipate heat and protect the material against early seizure [57,58]. The maximum depth of the friction path was determined. It was observed that the depth for the reference sample is 447.1 μm, while the addition of graphite, regardless of the weight fraction and particle size, significantly reduces the depth to approximately 65–75 μm. Only the sample containing 5% graphite by weight with a size below 10 μm has a depth of 194.2 μm, which confirms the results of insufficient reduction in the friction coefficient and a relatively large volume loss of the sample.

**Figure 10:**
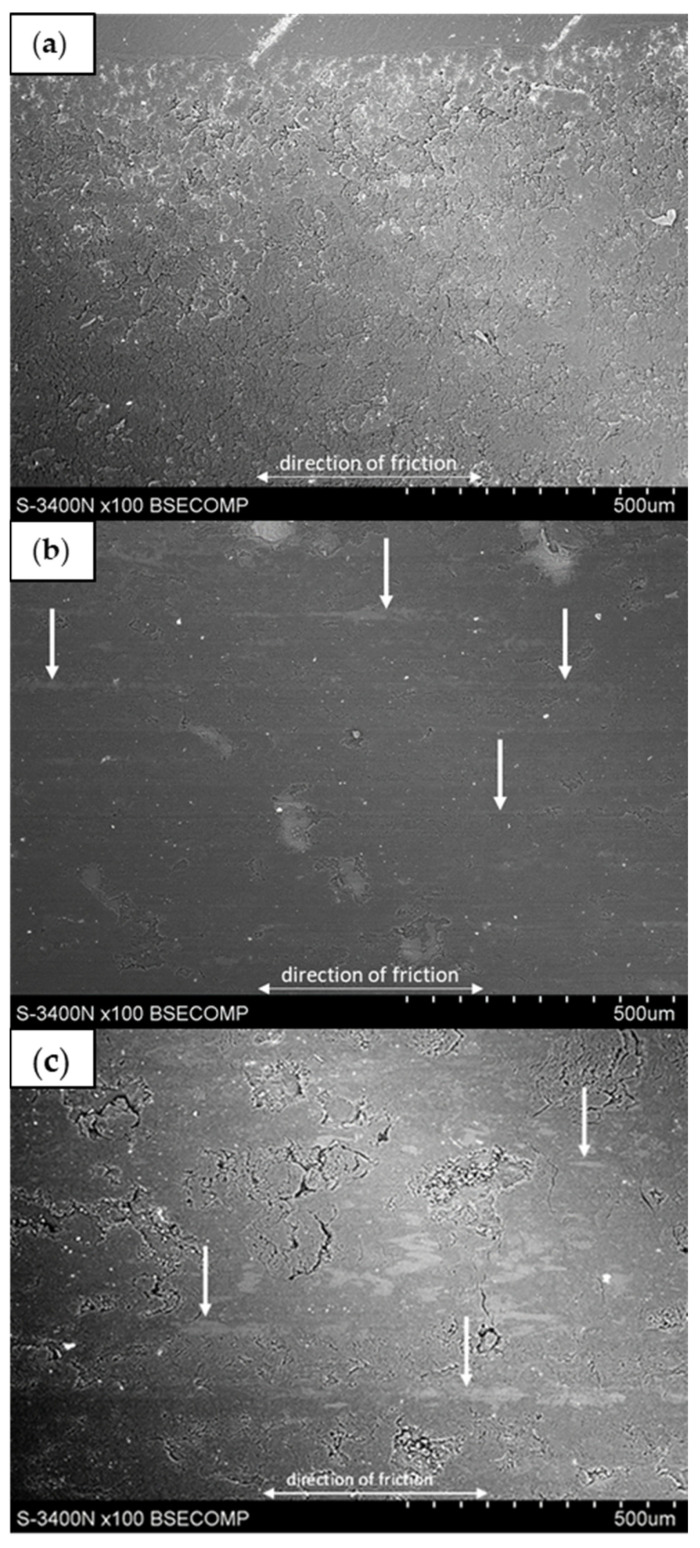
SEM micrographs of samples after sliding wear testing at room temperature: (**a**) reference sample; (**b**) G10_15; (**c**) G45_15.

At room temperature, the reference sample and graphite-filled composite samples showed typical abrasive wear behavior. The unfilled epoxy resin sample (reference) (Figure 10a) is characterized by numerous microcracks, mostly perpendicular to the sliding direction, which suggests the presence of a fatigue mechanism described in the literature [59,60,61,62], where plastic deformation of the resin occurs and particles are separated by delamination.

**Figure 11:**
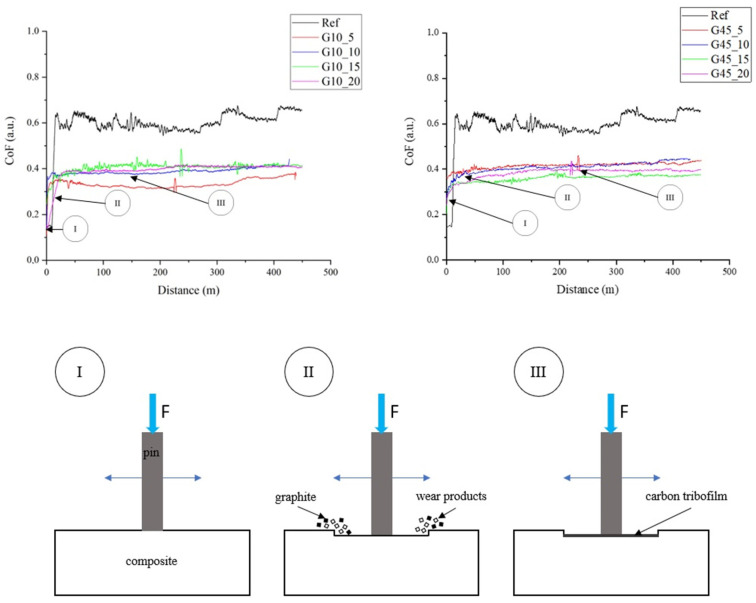
Mechanism of formation of the tribofilm on the composite surface.

Figure 11 shows a diagram illustrating the formation of a carbon tribofilm on the composite surface during friction. In the first stage (I) of technical dry friction, the composite comes into contact with the steel counter sample. In the second stage (II), the friction coefficient increases. The composite wears out, and the material is gradually removed. In the last stage (III), the friction coefficient stabilizes because a carbon tribofilm is formed between the materials, limiting abrasive wear.

The obtained tribological test results indicate that the addition of graphite has a beneficial effect on the tribological properties because it reduces the friction coefficient compared to the reference material (epoxy resin), and the tribofilm formed on the surface prevents abrasive wear and seizure. The formation of carbon tribofilm during sliding wear is a well-known mechanism, which has been described earlier in the literature [54,55,56]. Upon comparing the obtained results with literature data, it can be concluded that graphite is a cheap filler that reduces the friction coefficient by approximately 30–40%. Epoxy composites with the addition of molybdenum disulfide give [63] a reduction in the friction coefficient by 65%, with the addition of graphene by 60–90% [64], with the addition of graphene plates by approximately 50% [65] and with the addition of nanotubes and fullerenes by approximately 26–38% [66]. The observed mass and volume loss of the composite is largely due to the structural degradation of the resin caused by plastic deformation of the matrix and thermal decomposition. An increase in temperature at the interface of materials during friction leads to degradation of the polymer material by the adhesive wear mechanism, which often includes polymer bond breakage [67,68,69].

## 4. Conclusions

The proposed methodology for the production of samples based on liquid epoxy resin is effective and allows the production of homogeneous materials with low porosity. 

The tests carried out on composites with the addition of flake graphite allowed the following conclusions:

- Dynamic viscosity of the epoxy resin at the level of 600 cP with a gelation time of approximately 30 min (at a temperature of 20 °C) prevents the sedimentation process of graphite particles with a particle size below 45 μm and a weight addition above 5%;
- With the increase in the addition of flake graphite in the liquid epoxy resin, the ease of degassing of the system decreases, and the graphite content above 15% by weight leads to the formation of porosity in the material;
- The addition of harder graphite particles to the epoxy resin produces a synergistic effect of increasing the hardness of the composite by approximately 10%;
- The addition of flake graphite has a negative effect on the flexural strength, which decreases by approximately 20–30% compared to the unfilled epoxy resin. The increase in graphite content increases the tendency to create porosity inside the material, which has an adverse effect on the mechanical properties;
- There are no significant differences in the friction coefficient between graphite with a particle size below 10 μm and graphite with a particle size below 45 μm;
- Producing self-lubricating composites by adding flake graphite to epoxy resin is possible. The friction coefficient after adding graphite with a particle size below 45 μm allows the friction coefficient to be reduced by over 30% (from a value of 0.6 for unfilled resin to a value of 0.4 for composites). An additional advantage of adding graphite is a significant reduction in the abrasive wear of the material because graphite, due to friction, creates a carbon tribofilm on the surface of the material, providing protection against rapid wear;
- The developed sliding composite can be successfully used in the production of bearings, guides, slides, sleeves and other elements in the machinery industry, household appliances, vehicle components, etc.;
- Sliding composites based on resins with the addition of graphite are a low-cost material that allows for unit and mass production, which gives an advantage over traditional sliding materials, the unit production of which is unprofitable due to high tooling costs, e.g., the cost of producing an injection mold. Additionally, the geometry of products cast from resins allows for more complex geometry than that obtainable in the process of injection molding, extrusion or machining. The limitations of sliding composites based on liquid resins are the longer production time and problems related to the sedimentation of the filler and the porosity of the material.

Taking into account all recorded technological parameters and test results, it can be concluded that the most favorable series of samples are those containing 10% graphite by weight, both with a particle size below 10 μm and graphite with a particle size below 45 μm. The use of a smaller graphite addition may lead to partial sedimentation of the filler (due to insufficient viscosity of the system) and to difficulties in breaking in the material and stabilizing the friction coefficient, while a higher weight addition of graphite leads to numerous difficulties with degassing the system and the formation of porosity and inhomogeneity of the composite.

## Figures and Tables

**Table 1:** Sample compositions with designations.

Sample	Weight Addition, [%]	
	Graphite < 10 μm	Graphite < 45 μm
reference ^1^	0	0
G10_5	5	0
G10_10	10	0
G10_15	15	0
G10_20	20	0
G45_5	0	5
G45_10	0	10
G45_15	0	15
G45_20	0	20

^1^ Unfilled cured epoxy resin.

**Table 2:** Quantitative share of graphite particles in the cross-section of the epoxy composite filled with graphite.

Sample	Share of the Area, [%]
reference	0.00
G10_5	4.73
G10_10	8.22
G10_15	13.84
G10_20	19.77
G45_5	3.62
G45_10	8.66
G45_15	13.20
G45_20	19.27

**Table 3:** Results obtained in a three-point bending test in accordance with ISO 178 standard.

Sample	Flexural Strength, [MPa]	Standard Deviation	Young’s Modulus, [GPa]	Standard Deviation
reference	91.37	7.51	3.20	0.10
G10_5	63.36	5.17	2.90	0.18
G10_10	62.38	1.54	2.86	0.22
G10_15	57.26	3.17	3.43	0.52
G10_20	44.24	7.07	3.62	0.56
G45_5	55.41	2.80	2.56	0.04
G45_10	54.88	1.62	3.21	0.20
G45_15	52.81	3.81	3.42	0.48
G45_20	49.37	1.56	3.66	0.21

**Table 4:** Dynamic coefficient of friction and volume loss of the tested composites.

Sample	Dynamic Coefficient of Friction, μ	Standard Deviation of μ	Volume Loss, [cm^3^]	Mass Loss, [g]	Maximum Depth, [μm]
reference	0.60	0.08	0.0102	0.0116	447.1
G10_5	0.33	0.02	0.0003	0.0003	62.4
G10_10	0.39	0.02	0.0002	0.0002	76.2
G10_15	0.41	0.02	0.0003	0.0003	64.3
G10_20	0.39	0.04	0.0001	1 × 10^−4^	74.5
G45_5	0.42	0.01	0.0004	0.0005	65.8
G45_10	0.41	0.02	0.0003	0.0003	67.7
G45_15	0.36	0.02	0.0002	0.0002	65.9
G45_20	0.38	0.02	0.0001	0.0001	72.1

## Data Availability

Data are contained within the article.
